# Evaluation of pathogenesis caused in cattle and guinea pig by a *Mycobacterium bovis *strain isolated from wild boar

**DOI:** 10.1186/1746-6148-7-37

**Published:** 2011-07-12

**Authors:** Virginia Meikle, María V Bianco, Federico C Blanco, Andrea Gioffré, Sergio Garbaccio, Lucas Vagnoni, Julio Di Rienzo, Ana Canal, Fabiana Bigi, Angel Cataldi

**Affiliations:** 1 Institute of Biotechnology, Hurlingham, Argentina; 2 Institute of Pathobiology, Center for Veterinary and Agricultural Research, National Institute of Agricultural Technology (INTA), Hurlingham, Argentina; 3 Center for Veterinary and Agricultural Research, National Institute of Agricultural Technology (INTA), Hurlingham, Argentina; 4 School of Agricultural Sciences National University of Córdoba, Argentina; 5 School of Veterinary, National University of Litoral, Esperanza, Argentina

**Keywords:** boars, tuberculosis, bovines, Mycobacterium bovis

## Abstract

**Background:**

In many regions of the world, wild mammals act as reservoir of *Mycobacterium bovis*, a situation that prevents the eradication of bovine tuberculosis. In order to observe whether a strain isolated from a wild boar, previously tested as highly virulent in a mice model, is also virulent in cattle, we performed cattle experimental inoculation with this strain

**Results:**

Groups of Friesian calves were either infected with the wild boar strain *M. bovis *04-303 or with the bovine strain NCTC10772 as a control. We found that antigen-specific IFN-γ release in whole blood samples occurred earlier in animals infected with *M. bovis *04-303. Both *M. bovis *strains resulted in a positive skin test, with animals infected with the wild boar isolate showing a stronger response. These results and the presence of more severe organ lesions, with granuloma and pneumonic areas in cattle demonstrate that the wild boar isolate is more virulent than the NCTC10772 strain. Additionally, we tested the infectivity of the *M. bovis *strains in guinea pigs and found that *M. bovis *04-303 had the highest pathogenicity.

**Conclusions:**

*M. bovis *strains isolated from wild boars may be pathogenic for cattle, producing TB lesions.

## Background

*Mycobacterium bovis *has an extremely broad host range which makes bovine tuberculosis (bTB) difficult to eradicate worldwide. Management policies to control infections such as bTB must consider the main reservoirs in each region of interest.

Wildlife reservoirs are different all around the world. In Great Britain, Eurasian badgers (*Meles meles*) constitute the main reservoir [1], whereas in France, in an outbreak in wildlife, red deer (*Cervus elaphus*) were reported to be frequently infected with the same circulating *M. bovis *strain as that affecting nearby cattle herds [2]. In New Zealand, brushtail possums (*Trichosurus vulpecula*) are the main wildlife vectors and self-sustaining maintenance hosts of bTB, and thus play a role analogous to that of badgers in Great Britain [3]. In the US, white-tailed deer constitute an important reservoir of *M. bovis *in free-living wildlife [4]. Tuberculosis has also been observed in stoats, hares, and rabbits, but the level of infection recorded in their populations indicates that these species are unlikely to spread the disease to other animals and are hence not involved in the transmission of TB to livestock [3].

The concept that the wild boar constitutes a bTB reservoir host in Spain has been compiled by Naranjo et al. [5]. Strong evidence supporting this concept includes: (i) the presence of *M. tuberculosis *complex genotypes common to wild boars, domestic and wild animals and humans, (ii) the high prevalence of *M. bovis *observed among wild boars in fenced estates in complete absence of contact with domestic livestock and other wild ungulates, (iii) the bTB lesions frequently seen in thoracic lymph nodes and lungs, which suggest that respiratory infection and excretion may occur, and (iv) the extensive tuberculous lesions in more than one anatomical region found in a high proportion of juvenile wild boars that probably represents the main source of mycobacterial excretion [6,7]. In Argentina the wildlife species that may constitute a reservoir of animal tuberculosis are still unknown. However, in Argentina, wild boars (*Sus scrofa*) are present in large numbers, raising the possibility that wild boars constitute a reservoir for bovine tuberculosis. In 2006, two *M. bovis *isolates were obtained from wild boars killed in a hunting area. Experiments in mice with these isolates have shown a high virulence pattern with intense splenomegaly and extensive granuloma formation in lungs. In addition, survival of these animals was shorter when compared to animals infected by *M. bovis *reference strain AN5 and other strains from Argentina [8]. The spoligotype of one of these isolates matched the most common spoligotype of *M. bovis *isolated from infected cattle in Argentina [9], thus suggesting transmission between species. The risk of transmission of infection between a wildlife population and domestic animals needs to be determined by the epidemiology of the disease, the ecology of the host and the pathophysiology of the wild life strain in cattle. The aim of this work was to study and compare the infectivity and the immune response elicited in cattle by an *M. bovis *isolated strain from wild boars with those of a *M. bovis *strain isolated from cattle, the natural host.

## Methods

### Bacterial strains and culture media

*Mycobacterium bovis *NCTC 10772 is a bovine lymphnode isolate (http://www.straininfo2.ugent.be/culture/99859/catalog;jsessionid=C2B6AA33B1E3024D2889F173B08BDBF8) and *M. bovis *04-303 is an isolate from wild boar tuberculous lesions [8]. *M. bovis *04-303 has been previously characterized by Aguilar León et al. [8]. Both isolates were grown at 37°C on Middlebrook 7H9 (BD, USA) liquid medium plus 0.5% Tween 80 enriched with 0.4% pyruvic acid, and 1% albumin dextrose complex (ADC). Viable bacteria in the inocula were enumerated with the Live/Dead BacLight™ Bacterial Viability kit (Invitrogen, Molecular Probes, Carlsbad, California).

### Infection and sampling

Eight uninfected healthy Fresian calves of approximately 3 months of age were obtained from a TB-free herd (TB-free for the last five years). These calves were negative for the tuberculin skin test and showed absence of an *in vitro *gamma interferon (IFN-γ) response to both avian tuberculin PPD (PPDA) and bovine tuberculin PPD (PPDB). Animal experimentations were performed inside the biosafety facilities of the National Institute of Agricultural Technology (INTA), Argentina, in compliance with the regulations of the Ethical Committee of INTA and authorized by the National Service of Agricultural and Food Health and Quality (SENASA). Calves were intratracheally inoculated with 10^7 ^colony forming units (CFU) of *M. bovis *strains (four animals with NCTC 10772 and four animals with 04-303) as described previously [10]. Heparinized blood samples were taken at 15, 30, 60, 90, 120 and 150 days after inoculation (dpi), whereas nasal swabs were taken at 15, 30, 60, 90 and 120 dpi. The disease status of the animals was examined at post-mortem by the presence of lesions typical of bTB in lungs, liver, and retropharyngeal, mediastinal, and tracheobronchial lymph nodes. Tissue samples were processed for bacterial culture in Stonebrink medium as described elsewhere [11]. Cultures were processed by Ziehl-Neelsen staining when colonies appear and IS *6110*-PCR was performed on DNA from colonies to confirm its identity as *M. tuberculosis *complex.

### DNA extraction from swabs and PCR

Nasal swabs were collected using 50-cm long sterile swabs. Swabs were washed with 20 ml of sterile PBS in 50 ml centrifuge tubes. These were centrifuged at 4500 g for 20 min. The pellets were resuspended in 400 μl of 1 x Tris-EDTA (TE) buffer. DNA was prepared as described by Van Soolingen et al. [12]. The oligonucleotides 5'-cgtgagggcatcgaggtggc-3' and 5'-gcgtaggcgtcggtgacaaa-3' specific for a 245-bp IS *6110 *fragment were used. Touch-down amplification was performed with an initial step of 96°C for 3 min, followed by eight cycles of 96°C for 1 min, annealing temperatures starting at 72°C for 1 min (decreasing by 1°C/cycle), and extension at 72°C for 1 min [13]. This step was followed by 30 cycles of 96°C for 1 min, 65°C for 1 min, 72°C for 2 min, and extension at 72°C for 8 min.

### Gamma Interferon (IFN-γ) release assay

Heparinized blood samples were dispensed in 200 μl aliquots into individual wells of a 96-well plate. Wells contained whole blood plus 20 μg/ml *M. bovis *PPD (Prionics), 20 μg/ml *M. avium *PPD (Prionics), or 4 μg/ml of ESAT6 or CFP10 recombinant antigens. Blood cultures were incubated for 18 h, and plasma was harvested and stored at -80°C. IFN-γ concentrations in stimulated plasma were determined using a commercial ELISA-based kit (Bovigam™; Prionics). Absorbance of standards and test samples were read at 450 nm. The optical density (OD) for the PBS controls, which was usually approximately 0.1 OD units, was used to normalize individual readouts and to calculate optical density indexes (ODIs), where the results obtained by antigen stimulation were divided by the results of the PBS-stimulated cultures. Duplicate samples for individual antigens were analyzed. The interaction between time (days post infection, dpi) and strain (04-303 and NCTC 10772) was significant (p < 0.001) for all antigens. To analyze those interactions, a multiple comparison of treatment means was performed using DGC procedure 22 at a significance level of 0.05. This method was preferred because it prevents the occurrence of overlapping groups of means, and thus facilitates interpretation.

### Tuberculin testing

All animals were tested for skin tuberculin test (DTH) both before the *M. bovis *challenge and three months after the challenge. Animals were intradermally injected with 0.1 ml of PPDB and the thickness of the caudal fold tuberculin skin test were measured using calipers, and measured again 72 h after injection. PPDB (50,000 IU) was obtained from the National Service of Agricultural and Food Health and Quality (SENASA, Buenos Aires, Argentina).

### Guinea pig virulence testing

Groups of five guinea pigs (females of 300-350 g) were intratracheally inoculated with 10^3 ^CFU of either *M. bovis *NCTC 10772 or *M. bovis *04-303 strains. Survival was followed for 110 days. After death or sacrifice the lesions were observed and recorded. DTH testing was performed in surviving animals at 40 dpi by intradermal injection at the back with 0.3 μg and 1 μg of PPDB and PBS as a control. The induration diameter was read at 72 h.

### Histopathology: cattle and guinea pigs

Lungs, and retropharyngeal, mediastinal, and tracheobronchial lymph nodes in all cases and any other organ with lesions compatible with TB were collected and in cattle and guinea pigs and were fixed in 10% formaldehyde, sectioned and embedded in paraffin. Then, 5 μm-thick sections were stained with hematoxylin and eosin for histopathological examination. References were classified to a degree of I to IV according to Wangoo's criteria [14].

### Statistical analysis

To analyze the ODI results, a mixed model was fit for each antigen. In all cases, a correlation structure was estimated to take into account the correlation between the observations made in the same animal. All models included a variance function to take into account variance dependence on fitted values. Models were fit using the *nlme *R library through a user-friendly interface implemented in InfoStat [15,16].

## Results

### Experimental infection of bovines and surveillance of *M. bovis *infection by PCR in nasal swabs and blood samples

In nasal swabs, at 15 dpi, PCR was positive in two out of the four animals infected with *M. bovis *04-303 and in two out of the four animals infected with *M. bovis *NCTC 10772. At 30 dpi, PCR was positive in one animal infected with *M. bovis *04-303 and in three animals infected with *M. bovis *NCTC 10772. After this time, the number of positive samples increased at 60 dpi: three animals of each group were positive and at the end of the experiment, the presence of IS *6110 *was detected in all infected animals (Table 1). In blood samples, the number of *M. bovis *04-303-infected animals positive to PCR was higher than that of *M. bovis *NCTC 10772-infected ones. At later times, the results were variable, probably due to the low level of *M. bovis *DNA concentration in blood.

**Table 1:** IS *6110*-PCR in nasal swabs and blood samples

Animal	Strain	IS6110-PCR Nasal swab/blood sample					
		**0 dpi**	**15 dpi**	**30 dpi**	**60 dpi**	**90 dpi**	**120 dpi**

1		-/-	-/+	-/+	+/-	+/+	+/-
		
2	*M. bovis*	-/-	-/-	-/+	-/+	+/-	+/-
		
3	04-303	-/-	+/+	+/+	+/+	+/-	+/-
		
4		-/-	+/-	-/+	+/+	+/+	+/+

5		-/-	+/-	-/-	-/-	+/-	+/-
		
6	*M. bovis*	-/-	-/-	+/-	+/+	+/-	+/-
		
7	NCTC 10772	-/-	-/-	+/+	+/+	+/-	+/+
		
8		-/-	-/-	+/+	+/+	+/+	+/+

Positive (+) and, (-) negative PCR amplification. dpi: days post-infection.

### IFN-γ response

**Figure 1:**
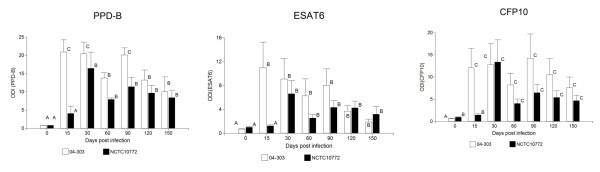
**Response of *M. bovis*-infected animals to antigens**. IFN-γ release in response to *M. bovis *antigens PPDB, ESAT6, CFP10. Results are expressed as ODIs. Columns with different letters are statistically different from each other. The mean values of ODIs plus their standard errors and significant differences are identified by letter coding.

The bovine immune cellular response against both strains was evaluated by IFN-γ production in response to PPDB and recombinant antigens (Figure 1). Although all infected animals developed strong antigen-specific IFN-γ responses to PPDB, animals inoculated with the *M. bovis *04-303 strain developed an early stronger response at 15 dpi. IFN-γ released by whole blood samples in response to ESAT6 and CFP10 followed a similar pattern, with a high response at 15 dpi in animals infected with 04-303 compared to those infected with the NCTC 10772 isolate. In the group infected with *M. bovis *04-303, the responses to PPDB at 60 dpi showed higher ODIs in the IFN-γ test than in the group infected with the NCTC 10772 strain.

### Delayed-type hypersensitivity response

The injection of *M. bovis *PPD resulted in negative tuberculin skin test reactions in all animals before the *M. bovis *infection. All animals were positive in the single intradermal tuberculin test at 90 dpi and *M. bovis *04-303-inoculated cattle showed a stronger reaction than *M. bovis *NCTC 10772-inoculated ones (p < 0.05) by Student' t-test.

### Pathological, histopathological tests and microbiological studies after challenge

Animals were killed after 5 months of infection. Typical tuberculosis lesions were found in three *M. bovis *04-303-infected cattle. In this experimental group, lesions were granulomatous and pneumonic. In the three animals, lesions were localized in the left and right apical lobe lung, and in mediastinal lymph node and liver. The remaining animal showed no lesions. In the group inoculated with NCTC 10772 only one animal showed granulomatous lesions in the retropharyngeal lymph nodes (data not shown). The histopathological analysis revealed that while animals infected with *M. bovis*-04-303 showed type II and III granulomas in lung and lymphnodes in two animals and even type IV in another animal, no granulomas were observed in *M. bovis *NCTC 10772-infected cattle. In *M. bovis *04-303 but not in *M. bovis *NCTC 10772-infected cattle also presented pneumonic areas and bronchiole infiltration (data not shown).

*M. bovis *culture was positive in lymph nodes and lungs in both groups of animals. In general, Ziehl-Neelsen staining and PCR from colonies were positive when culture was positive (Table 2). In many cases, *M. bovis *was recovered from cultures of organs where no macroscopic or microscopic lesions were observed.

**Table 2:** Microbiological culture, Ziehl Neelsen staining and PCR amplification from cultures

Group	Animal	Organ	GrossLesion	Culture	ZN	PCR
	1	retropharyngeal LN	N	N	N	N
		
		mediastinal LN	N	N	N	N
		
		tracheobronchial LN	N	N	N	N
		
		lung	N	N	N	N
	
	2	retropharyngeal LN	N	N	N	N
		
		mediastinal LN	N	N	N	N
		
04-303		tracheobronchial LN	N	P	P	P
		
		lung	P	P	P	P
	
	3	retropharyngeal LN	N	N	N	N
		
		mediastinal LN	N	N	N	N
		
		tracheobronchial LN	N	P	P	P
		
		lung	P	P	P	P
		
		liver	P	P	P	P
	
	4	retropharyngeal LN	N	P	P	P
		
		mediastinal LN	P	P	P	P
		
		tracheobronchial LN	N	P	P	P
		
		lung	P	P	P	P

	5	retropharyngeal LN	P	P	P	P
		
		mediastinal LN	N	N	N	N
		
		tracheobronchial LN	N	N	N	N
		
		lung	N	P	P	P
	
	6	retropharyngeal LN	N	N	N	N
		
		mediastinal LN	N	P	P	P
		
NCTC 10772		tracheobronchial LN	N	P	P	P
		
		lung	N	N	N	N
	
	7	retropharyngeal LN	N	N	N	N
		
		mediastinal LN	N	P	P	P
		
		tracheobronchial LN	N	N	N	N
		
		lung	N	N	N	N
	
	8	retropharyngeal LN	N	N	N	N
		
		mediastinal LN	N	N	N	N
		
		tracheobronchial LN	N	N	N	N
		
		lung	N	N	N	N

LN: lymphnode

N: negative

P: positive.

### Virulence testing of both *M. bovis *strains in guinea pigs

**Figure 2:**
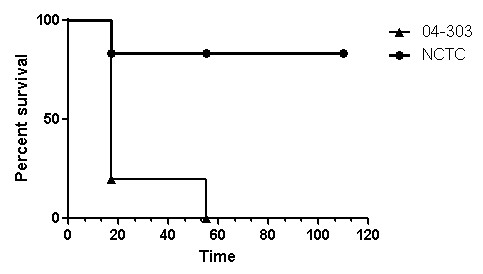
**Survival curve of guinea pigs infected with *M. bovis *04-303 or *M. bovis *NCTC 10772**. Both curves were statistically different by the Mantel-Cox test (p = 0.014).

In order to confirm the results observed in cattle, groups of guinea pigs were infected with either *M. bovis *04-303 or *M. bovis *NCTC 10772 and the survival rate was used as a main parameter of virulence. In the group infected with *M. bovis *04-303, four animals died at 17 dpi and one animal died at 55 dpi. In the case of guinea pigs inoculated with NCTC 10772, two animals died before 30 dpi, whereas the remaining animals survived until the end of the experiment. A survival curve is shown in Figure 2. DTH was performed with PPDB at 40 dpi in surviving animals. Induration reaction after intradermal inoculation of 0.3 μg of PPDB was detected. In animals infected with *M. bovis *04-303, the reaction was 3 mm, while in those infected with *M. bovis *NCTC 10772 it was 0.7 ± 0.19 mm. When 1.0 μg of the antigen was intradermally inoculated, the induration of animals infected with *M. bovis *04-303 was 6.5 mm and in those infected with *M. bovis *NCTC 10772 was 1.8 ± 0.02. At necropsy, animals infected with *M. bovis *04-303 that died at 17 dpi presented small purulent granulomas in lungs, while the one that died at 55 dpi showed large granulomas (data not shown).

The histopathological analysis of three out of four animals infected with NCTC 10772 showed extensive pneumonic areas with incipient type I or II granulomas. The other animal showed a less extended pneumonic area with cell infiltration in bronchioles. At macroscopic examination, two animals showed granulomatous lesions and the two other no apparent lesions (data not shown). Four out of five of the animals experimentally infected with *M. bovis *04-303 had extensive pneumonic areas covering almost all the lung surface with incipient granulomas. Most of these animals died at 17 dpi. Another animal presented small areas with cell infiltration and pneumonia. At macroscopic examination, three of the animals that had died at 17 dpi did not present macroscopic TB lesions (data not shown). The animal that had died at 55 dpi presented numerous lesions.

## Discussion

In a previous study carried out in a murine model of tuberculosis, different *M. bovis *strains showed wide virulence variability [8]. In that work, the strain *M. bovis *04-303 isolated from a wild boar in Argentina showed to be the most virulent, killing the mice soon after infection and producing high lung bacillary loads and extensive pneumonia accompanied by massive necrosis. Similar results of survival and bacillary loads were obtained in another work with the hypervirulent *M. tuberculosis *Beijing strain 9501000 [17]. The Beijing strain, however, did not produce necrosis as massive as that observed with *M. bovis *04-303 [8].

In this study, we assessed the virulence of the wild boar *M. bovis *isolate and compared it with that of the *M. bovis *NCTC 10772 in a cattle model of infection. This *M. bovis *NCTC 10772 collection strain was selected as a virulent control strain based on previous findings demonstrating that this strain is capable of replicating in mouse organs [10] and producing lesions compatible with tuberculosis in cattle after intratracheal inoculation of 10^9 ^CFU (data not shown). In our conditions, experimental inoculation produced fewer lesions as observed by other authors [18,19]. We think that this situation is caused by non tuberculous mycobacteria exposure and helminth infections that provoked an increased resistance to *M. tuberculosis *complex pathogenesis, as postulated by other authors [20].

Detectable immune responses and reactivity in skin tuberculin test were observed in both groups of animals after the bacterial infection and throughout the study, indicative of bTB infection. As expected, all the infected animals were IS *6110 *PCR-positive in nasal swabs at the end of the experiment. Animals infected with *M. bovis *04-303 became positive earlier after inoculation, whereas the *M. bovis *NCTC 10772-infected animals were positive at 30 dpi. In our knowledge this is the first report on the presence of *M. bovis *in bovine blood and nasal secretions along the infective cycle. We have previously observed that PCR results to detect *M. bovis *are variable from animal to animal and in the same animal along time [21]. There are scarce papers about the presence of mycobacteria in blood from cattle. The intermittent *M. avium *susbp *paratuberculosis *bacteremia has been reported [22,23]. These and our results suggest that mycobacteria may dispersed in blood, perhaps on phagocytic cell, as proposed by Juste et al [24]. The detection of *M. bovis *in nasal swab is expected as it is respiratory pathogen and it has been reported previously [13,21,25-27].

ESAT6 elicited high levels of IFN-γ at 15 dpi in animals infected with *M. bovis *04-303 and the levels were statistically significantly different compared to those of animals infected with *M. bovis *NCTC 10772. In the late infection, the stimulation of blood with either ESAT6 or CFP10 recombinant antigens did not show differences in IFN-γ production between animal groups.

Therefore, the presence of lesions compatible with bTB in lungs of infected animals and the early detection of bacilli in blood samples and swabs demonstrate that the *M. bovis *strains used in this study effectively infected bovines. However, the experimental infection of cattle with the *M. bovis *04-303 strain causes lesion in lungs, while infection with *M. bovis *NCTC 10772 causes lesion only in lymphnodes. The short time period studied and the lower virulence of *M. bovis *NCTC 10772 could explain the reduced number of animals with lesion in the group infected with this strain. In addition, the outcome of guinea pigs intratracheally inoculated supports the conclusion of the higher virulence of the strain from wild boar.

The results of this study suggest that tuberculosis infection of wild boars is a risk factor associated with the propagation of *M. bovis*. This hypothesis is supported by the study of Aguilar et al. [8], who, by using a model of TB in mice, demonstrated the hypervirulent behaviour of two *M. bovis *strains isolated from wild boars of Argentina, one of which was the *M. bovis *04 303 strain. Wild boars are numerous in Argentina, and it is postulated that the presence of a reservoir of bTB in these animals may frustrate efforts to reduce the incidence of bTB by the test and slaughter approach. If this is the case, ways to reduce the burden of disease in these animals will need to be contemplated, like that being done in badgers in Europe and in brushtail possums in New Zealand [28].

## Conclusions

This report highlights the need to control the infection in wildlife reservoirs of bovine tuberculosis and the importance of research on the interaction between infected wild boars and bovine tuberculosis in cattle.

## Authors' contributions

VM: Participated in the infection, sampling and necropsy of animals, in DNA extraction from swabs followed by PCR. She performed the gamma Interferon (IFN-γ) release assay, Tuberculin testing, and Guinea Pig virulence testing. She wrote the paper. MVB: Performed in the inoculum preparation and helped in the sampling for the necropsy and Guinea Pig virulence testing. She did the histopathology analysis. FCB: Participated in infection and sampling of animals. AG: Helped in analyzing the results and drafting the manuscript. SG: Participated in infection and sampling, Tuberculin testing, and necropsy. LV: carried-out the infection and necropsy of animals. JDiR: participated in the study design and made the statistical analyses. AC: directed the analysis of the histopathology of cattle and guinea pigs. FB: participated in the design of the study and helped preparing the manuscript. AC: Designed the study, analyzed the results and prepared the manuscript. All authors read and approved the final manuscript.
